# Prevalence of *Campylobacter* Species in Adult Crohn's Disease and the Preferential Colonization Sites of *Campylobacter* Species in the Human Intestine

**DOI:** 10.1371/journal.pone.0025417

**Published:** 2011-09-23

**Authors:** Vikneswari Mahendran, Stephen M. Riordan, Michael C. Grimm, Thi Anh Tuyet Tran, Joelene Major, Nadeem O. Kaakoush, Hazel Mitchell, Li Zhang

**Affiliations:** 1 School of Biotechnology and Biomolecular Sciences, University of New South Wales, Sydney, Australia; 2 Gastrointestinal and Liver Unit, The Prince of Wales Hospital, Sydney, Australia; 3 Faculty of Medicine, University of New South Wales, Sydney, Australia; 4 St George Clinical School, University of New South Wales, Sydney, Australia; Charité, Campus Benjamin Franklin, Germany

## Abstract

**Introduction:**

Crohn's disease (CD) and ulcerative colitis (UC) are the two major forms of inflammatory bowel disease (IBD). A high prevalence of *Campylobacter concisus* was previously detected in paediatric CD and adult UC. Currently, the prevalence of *C. concisus* in adult CD and the preferential colonization sites of *Campylobacter* species in the human intestine are unknown. In this study, we examined the prevalence of *Campylobacter* species in biopsies collected from multiple anatomic sites of adult patients with IBD and controls.

**Methods:**

Three hundred and one biopsies collected from ileum, caecum, descending colon and rectum of 28 patients IBD (15 CD and 13 UC) and 33 controls were studied. Biopsies were used for DNA extraction and detection of *Campylobacter* species by PCR-sequencing and *Campylobacter* cultivation.

**Results:**

A significantly higher prevalence of *C. concisus* in colonic biopsies of patients with CD (53%) was detected as compared with the controls (18%). *Campylobacter* genus-PCR positivity and *C. concisus* positivity in patients with UC were 85% and 77% respectively, being significantly higher than that in the controls (48% and 36%). *C. concisus* was more often detected in descending colonic and rectal biopsies from patients with IBD in comparison to the controls. *C. concisus* was isolated from patients with IBD.

**Conclusion:**

The high intestinal prevalence of *C. concisus* in patients with IBD, particularly in the proximal large intestine, suggests that future studies are needed to investigate the possible involvement of *C. concisus* in a subgroup of human IBD. To our knowledge, this is the first report of the association between adult CD and *C. concisus* as well as the first study of the preferential colonization sites of *C. concisus* in the human intestine.

## Introduction

*Campylobacter* species have been associated with various diseases in both animals and humans [1]. *Campylobacter jejuni* and *Campylobacter coli* are well established human pathogens, having been associated with a number of clinical conditions such as diarrhoea, abortion, septicaemia and Guillain-Barre syndrome [1]. Some other *Campylobacter* species including *Campylobacter concisus* have been considered as emerging human pathogens [2].

*C. concisus* is a curved Gram negative bacterium; with a single polar flagellum [3]. *C. concisus* was first isolated by Tanner *et al* in 1981 from human dental plague [4]. In a following-up study, Macuch and Tanner reported a higher isolation rate of *C. concisus* in patients at the initial stage of periodontitis in comparison to individuals with healthy gums [5].

Lately, *C. concisus* has been considered as an emerging human enteric pathogen [6]. Evidence that *C. concisus* may be an important human enteric pathogen has come from a number of recent studies reported that *C. jejuni* and *C. concisus* are the most commonly isolated *Campylobacter* species from diarrheal stool specimens [2], [7], [8], [9]. However, when Engberg *et al* compared the prevalence of *C. concisus* in 107 stool samples subjected to tests for enteric pathogens and in 107 age/sex matched healthy controls, they found that the prevalence of *C. concisus* in these two groups was not significantly different [9]. Furthermore, they found that *C. concisus* was more often isolated from children aged 0–9 years and individuals aged over 60 years as compared with other age groups. These results have led Engberg *et al* to conclude that *C. concisus* should be considered as a commensal bacterium and this bacterium may be an important opportunistic pathogen in individuals with compromised or immature immune systems [9].

In addition to periodontal and diarrheal diseases, recently *C. concisus* has been linked to inflammatory bowel disease (IBD). IBD is a chronic inflammatory condition of the gastrointestinal tract, with the two major forms being Crohn's disease (CD) and ulcerative colitis (UC). The inflammation in CD may occur anywhere along the gastrointestinal tract, however in UC the inflammation often occurs in colon and rectum [10]. The aetiology of IBD is currently unknown. It is understood that a complex interaction of a number of factors including host genetics, environment, immune system and intestinal microflora contributes to the development of IBD [10], [11], [12]. Despite strong evidence that the intestinal microbial flora plays a key role in the development of IBD, the exact causative agent (s) is still under investigation [11].

Previously, we detected a significantly higher prevalence of *C. concisus* by PCR in intestinal biopsies of children with CD (51%) as compared with the controls (2%) and isolated a *C. concisus* strain from intestinal biopsies of a child with CD [13]. In a later study, we detected high prevalence of *C. concisus* in stool samples of children with CD [14]. A recent study by Mukhopadhya *et al* reported a significantly higher prevalence of *C. concisus* detected by PCR in adult patients with UC as compared with the controls [15].

To date, the prevalence of *C. concisus* in adult patients with CD has not been investigated. Furthermore, no information is available regarding whether *Campylobacter* species preferentially colonize specific sites in the human intestine. In this study, we examined the prevalence of *Campylobacter* species in biopsies collected from four anatomic sites of intestines from adult individuals with normal intestinal histology and patients with IBD by PCR-sequencing and *Campylobacter* cultivation.

## Materials and Methods

### Ethics statement

Intestinal biopsies were obtained from colonoscopy procedures carried out at the Prince of Wales Hospital and the St George Hospital at Sydney, Australia. Ethics approval for this study was granted by the Ethics Committees of the University of New South Wales and the South East Sydney Area Health Service, Australia (HREC 09237/SESIAHS 09/078 and HREC08335/SESIAHS(CHN)07/48). Written informed consent was obtained from all subjects in this study.

### Study subjects and biopsy collection

Sixty-one study subjects, including 28 patients with IBD and 33 controls, were recruited from the Prince of Wales Hospital and the St George Hospital at Sydney, Australia. Among the 28 patients with IBD (15 CD and 13 UC), ten patients (six CD and four UC) were relapsed cases and the remaining eighteen patients were newly diagnosed IBD. Disease location and severity were scored according to the Montreal criteria [16]. The controls, either presenting with gastrointestinal symptoms including abdominal pain and constipation or undertaking a screening colonoscopic examination due to previous history of polyps or a family history of colonic cancer, had no macroscopic or microscopic intestinal inflammation.

Five biopsies were collected from each individual. In the case where macroscopic inflammation was present, biopsies were taken from the edge of the inflamed areas. Of the five biopsies collected from each individual, four biopsies collected from each of the four anatomic sites (ileum, caecum, descending colon and rectum respectively) were used for DNA extraction and detection of *Campylobacter* species by PCR. The additional biopsy collected from caecum was used for *Campylobacter* cultivation.

### DNA extraction from intestinal biopsies

Freshly collected intestinal biopsies were directly placed into cell lysis solution and DNA was extracted using the Puregene DNA Extraction kit (Gentra, Minneapolis, USA) according to the manufacturer's instructions.

### Detection of *Campylobacter* species in intestinal biopsies by *Campylobacter* genus-PCR

To detect all *Campylobacter* species, DNA extracted from intestinal biopsies were subjected to a nested *Campylobacter* genus-PCR. Bacterial 16S rRNA gene was first amplified from 200 ng of DNA extracted from intestinal biopsies using universal primers F27 and R1496 [17]. The thermal cycling conditions were 94°C for 10 minutes, followed by 35 cycles of 94°C for 10 seconds, 53°C for 10 seconds and 72°C for 1 minute. The PCR reaction volume was 25 µl. The PCR product was then purified using QIAquick PCR purification kit (Qiagen, Hilden, Germany). The purified PCR product (2 µl) was subjected to a *Campylobacter* genus-specific PCR using primers C418 and C1228 designed by Linton *et al* [18]. The thermal cycling conditions for the *Campylobacter* genus-specific PCR were 94°C for 5 minutes, followed by 35 cycles of 94°C for 30 seconds, 55°C for 30 seconds and 72°C for 30 seconds.

### *Campylobacter* species identification

All positive PCR products were sequenced using the BigDyeTM terminator chemistry (Applied Biosystems, Foster City, USA) and the sequencing mixture was analysed on DNA sequence analyser ABI3720 (Applied Biosystems, Foster City, USA). The obtained sequences were compared to gene sequences of known bacterial identities available in GenBank through the National Centre for Biotechonology Information (NCBI website (http://www.ncbi.nlm.nih.gov).

### *C. concisus* specific PCR

Three samples which had mixed sequences in the *Campylobacter* genus-PCR were subjected to a previously described *C. concisus* PCR to examine if *C. concisus* was present [14]. For the *C. concisus* PCR, DNA (50 ng) extracted from biopsies was subjected to the *Campylobacter* genus-PCR, then 1 µl of the *Campylobacter* genus-PCR product was subjected to *C. concisus* PCR as previously described [14].

### Cultivation of *Campylobacter* species from intestinal biopsies

One caecal biopsy collected from each individual was subjected to *Campylobacter* cultivation. The biopsy was spread on agar plates prepared using blood agar base no 2 supplemented with 6% sterile defibrinated horse blood, trimethoprim (10 µg/ml), and vancomycin (10 µg/ml). The plates were incubated under microaerophilic conditions generated by a *Campylobacter* gas generating system (Oxoid Limited, Hampshire, United Kingdom) for two days. A bacterial suspension was prepared from the culture plates and filtered through a 0.6 µM filter membrane (Millipore, Billerica, USA) onto a fresh agar plate and further incubated for additional two days.

Candidate colonies were subjected to microscopic examination of morphology, Gram staining, PCR targeting the 16S rRNA gene using primers F27 and R1649 and sequencing of the PCR products.

### GenBank Sequence Submission

All 16S rRNA gene sequences of the PCR products were submitted to GenBank.

### Statistical analysis

Fisher's exact test (two tailed) was used to compare the prevalence of *Campylobacter* species in patients with IBD and controls. Unpaired *t* test was used to compare the age of patients and controls. Statistical analysis was performed using GraphPad Prism 5 software (San Diego, CA).

## Results

### Clinical information of patients and controls

The average age of the patients with IBD and controls was 39±13 and 45±11 years old respectively. There were 12 male (43%) patients with IBD and 13 male in controls (39%). The age and sex between patients with IBD and controls were not statistically different.

A total of 301 biopsies (165 biopsies from 33 controls and 136 biopsies from 28 patients with IBD) were collected from four intestinal sites (ileum, caecum, descending colon and rectum) of patients with IBD and controls. Ileal biopsies were not available from two patients with CD, a caecal biopsy was not available from one patient with CD and a rectal biopsy was not available from an additional patient with CD. Both patients and controls did not receive antibiotics one month prior to colonoscopy.

All controls had normal intestinal histology. The Montreal classification of patients with IBD is summarized in Table 1.

**Table 1:** Montreal classification of patients with IBD

**Montreal classification (CD)**	**CD (n = 15)**
**L1**	7% (1/15)
**L2**	60% (9/15)
**L3**	33% (5/15)
**Montreal classification (UC)-Extent**	**UC (n = 13)**
Proctitis E1	8% (1/13)
Left sided UC E2	38% (5/13)
Extensive UC E3	54% (7/13)
**Montreal classification (UC)-Severity**	**UC (n = 13)**
Clinical remission S0	0
Mild UC S1	54% (7/13)
Moderate UC S2	46% (6/13)
Severe UC S3	0

### Prevalence of *Campylobacter* species in biopsies collected from different intestinal sites of individuals with normal intestinal histology

To examine the possible preferential colonization sites of *Campylobacter* species particularly *C. concisus* in the human intestine, DNA samples extracted from biopsies collected from four intestinal anatomic sites of 33 individuals with normal intestinal histology were subjected to *Campylobacter* genus-PCR. Among the 33 individuals examined, 48% (16/33) were positive for *Campylobacter* genus-PCR (an individual with at least one of the four intestinal biopsies collected from ileum, caecum, descending colon and rectum positive by the *Campylobacter* genus-PCR was considered *Campylobacter* genus-PCR positive). Of the 16 individuals who were positive by the *Campylobacter* genus-PCR, four individuals had one biopsy positive and 12 individuals had 2–4 biopsies positive by the PCR. *Campylobacter* genus-PCR positive rate in biopsies collected from ileum, caecum, colon and rectum were 27% (9/33), 30% (10/33), 27% (9/33), rectum 27% (9/33) respectively, with no statistical differences observed between sites. *Campylobacter* genus-PCR positive rate in male was 42% (5/12); with no statistical difference from that in females (52%, 11/21).

Sequencing of the positive PCR products yielded 503–766 bp sequences. The obtained sequences were used for identification of *Campylobacter* species. The similarities of 16S rRNA gene sequences between the *Campylobacter* genus-PCR products and the known *Campylobacter* species were 97%–100%. Five *Campylobacter* species were identified from biopsies collected from individuals with normal intestinal histology, including *C. concisus*, *Campylobacter showae*, *Campylobacter hominis*, *Campylobacter ureolyticus* and *Campylobacter hyointestinalis*. Among the 12 individuals who had multiple biopsies positive for the *Campylobacter* genus-PCR, single *Campylobacter* species was identified in eight individuals and two *Campylobacter* species were identified in the remaining four individuals.

Among the 33 individuals examined, 36% (12/33) of individuals were positive for *C. concisus,* 6% (2/33) of individuals were positive for *C. showae*, 9% (3/33) of individuals were positive for *C. hominis*, 6% (2/33) of individuals were positive for *C. ureolyticus* and 3% (1/33) of individuals were positive for *C. hyointestinalis*. For an individual to be classified as *C. concisus* positive, *C. concisus* had to be identified in at least one of the four biopsies collected. The same principle applied for the evaluation of the intestinal prevalence of the other *Campylobacter* species in this study.

*Campylobacter* species detected in biopsies collected from the four intestinal anatomic sites of individuals with normal histology is shown in Table 2. Ileal, caecal and colonic biopsies showed similar *C. concisus* positive rates and the rectum had a lower *C. concisus* positive rate; however the difference was not statistically significant. Given the low positive rate for the remaining four *Campylobacter* species, no statistical analysis was applied to compare the prevalence of these *Campylobacter* species in different sites of the intestines (Table 2).

**Table 2:** Detection of *Campylobacter* species in biopsies collected from four intestinal anatomic sites of individuals (n = 33) with normal intestinal histology by *Campylobacter* genus-PCR and sequencing*.

	Ileum	Caecum	Colon	Rectum
*C. concisus*	21% (7/33)	18% (6/33)	18% (6/33)	9% (3/33)
*C. showae*	3% (1/33)	3% (1/33)	3% (1/33)	6% (2/33)
*C. hominis*	3% (1/33)	6% (2/33)	3% (1/33)	9% (3/33)
*C. ureolyticus*	0	0	3% (1/33)	3% (1/33)
*C. hyointestinalis*	0	3% (1/33)	0	0

*Four biopsies, one each from ileum, caecum, descending colon and rectum of each individual, were examined. Identification of *Campylobacter* species was based on 97–100% similarity of the sequences of PCR products (503–766 bp) to the sequences of known *Campylobacter* species.

### Comparison of intestinal prevalence of *Campylobacter* species in patients with IBD and controls

**Figure 1:**
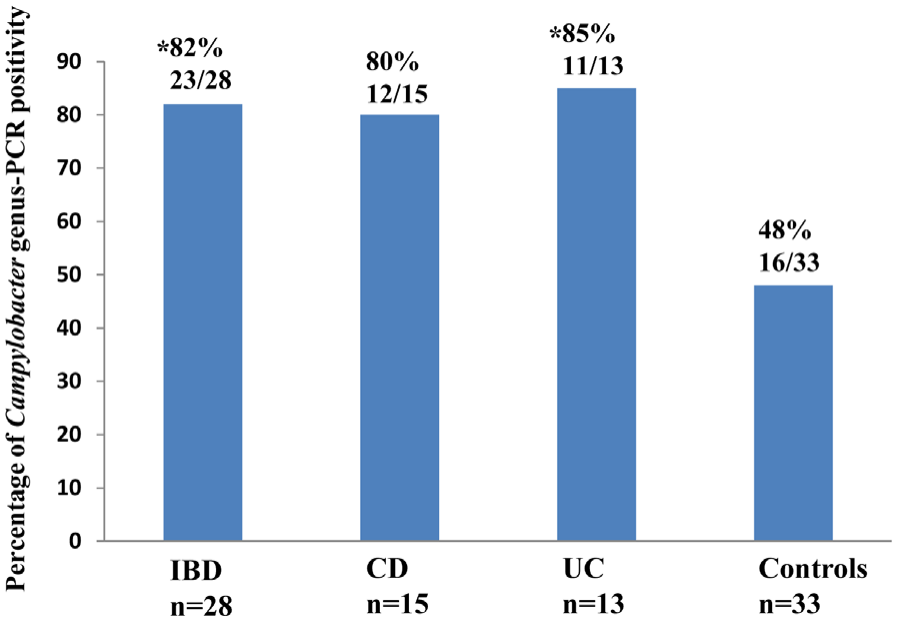
*Campylobacter* genus-PCR positivity in patients with IBD (CD and UC) and controls. Four biopsies collected from each individual were examined. A *Campylobacter* genus-PCR positive individual is an individual who had at least one intestinal biopsy positive for *Campylobacter* genus-PCR. *Significantly different as compared with the controls (*P*<0.05).

The above 33 individuals with normal intestinal histology were used as controls. *Campylobacter* genus-PCR positive rate in patients with IBD was 82% (23/28), which was significantly higher than that of the controls (48%, 16/33) (*P*<0.05). The *Campylobacter* genus-PCR positive rate was 80% (12/15) in patients with CD and 85% (11/13) in patients with UC; with the difference between UC group and the controls being statistically significant (*P*<0.05) and the difference between CD and controls being not statistically significant (Figure 1).

The prevalence of different *Campylobacter* species in patients with IBD and controls is shown in Table 3. Eight *Campylobacter* species were identified in intestinal biopsies collected from patients with IBD. *C. concisus* was detected in 68% (19/28) patients with IBD, which was significantly higher as compared with the controls 36% (12/33) (*P*<0.05). The *C. concisus* positive rates in patients with CD and UC were 67% (10/15) and 69% (9/13) respectively; the *C. concisus* positive rate between UC and controls was statistically different (*P*<0.05) and the difference between CD and the controls was not significantly different. The prevalence of the remaining seven *Campylobacter* species in patients with IBD and controls was not statistically different (Table 3).

**Table 3:** Percentage of *Campylobacter* species positivity in patients with IBD (CD and UC) and controls^@^.

	IBD (n = 28)	CD (n = 15)	UC (n = 13)	Controls (n = 33)
*C. concisus*	68% (19/28)*	67% (10/15)	69% (9/13)*	36% (12/33)
*C. showae*	11% (3/28)	7% (1/15)	15% (2/13)	6% (2/33)
*C. hominis*	7% (2/28)	7% (1/15)	8% (1/13)	9% (3/33)
*C. ureolyticus*	10% (3/28)	13% (2/15)	8% (1/13)	6% (2/33)
*C. hyointestinalis*	4% (1/28)	7% (1/15)	0	3% (1/33)
*C. rectus*	4% (1/28)	0	8% (1/13)	0
*C. jejuni*	4% (1/28)	7% (1/15)	0	0
*C. gracilis*	7% (2/28)	7% (1/15)	8% (1/13)	0

@A specific Campylobacter species positive individual is an individual who has at least one biopsy positive for the Campylobacter species listed in Table 3, detected by Campylobacter genus-PCR and sequencing. *Significantly higher as compared with the controls (P<0.05).

The *C. concisus* positive rate in relapsed CD was 67% (4/6), which was not significantly different as compared with the newly diagnosed CD cases (67%, 6/9). The *C. concisus* positive rate in relapsed UC was 75% (3/4), which was not significantly different from that of the new cases (67%, 6/9).

Of the three biopsies which showed mixed sequences by *Campylobacter* genus-PCR; two samples were positive and one was negative by *C. concisus* PCR.

### Comparison of prevalence of *C. concisus* in biopsies collected from different anatomic intestinal sites of patients with IBD and controls

Given that *C. concisus* was the only *Campylobacter* species showing statistical difference between patients with IBD and the controls in this study (Table 3), the prevalence of *C. concisus* in biopsies collected from ileum, caecum, colon and rectum of patients with IBD and controls was further compared and the results are shown in Table 4. Ileal biopsies collected from patients with IBD and controls showed similar *C. concisus* positivity. Caecal biopsies of patients with UC had a low *C. concisus* positive rate; however it was not statistically different from the other groups. *C. concisus* positive rate of colonic biopsies of patients with IBD was 43% (12/27), which was significantly higher compared to the controls (*P<0.05*). The *C. concisus* positivity in colonic biopsies of patients with CD and UC was 53% (8/15) and 31% (4/13) respectively; with patients with CD showing a statistically significant difference when compared to the controls (*P*<0.05). The *C. concisus* positivity in rectal biopsies of patients with IBD was higher than that of the controls; however it was not statistically different (Table 4).

**Table 4:** Detection of *C. concisus* in biopsies collected from four intestinal anatomic sites of patients with IBD (CD and UC) and controls^@^.

	IBD n = 28	CD n = 15	UC n = 13	Control n = 33
Ileum	23% (6/26)	23% (3/13)	23% (3/13)	21% (7/33)
Caecum	15% (4/27)	21% (3/14)	9% (1/13)	18% (6/33)
Colon	43% (12/28)*	53% (8/15)*	31% (4/13)	18% (6/33)
Rectum	26% (7/27)	21% (3/14)	31% (4/13)	9% (3/33)

@ Identification of C. concisus was based on Campylobacter genus-PCR and sequencing of the positive PCR products, except for three biopsy samples. The three biopsy samples showed mixed sequences by Campylobacter genus-PCR, therefore were further subjected to C. concisus PCR to examine the presence of C. concisus.

Biopsies collected from four intestinal anatomic sites were examined; ileal biopsies were not available from two patients with CD, caecal biopsy was not available from one patient with CD and rectal biopsy was not available from one patient with CD.

*Significantly higher in patients with IBD as compared with the controls (*P*<0.05).

### Prevalence of *C. concisus* in relation to Montreal classification of IBD

No significant differences were noted between the prevalence of *C. concisus* in patients with different Montreal classifications.

### Isolation of *C. concisus* from intestinal biopsies of patients with IBD and controls

*C. concisus* was isolated from intestinal biopsies of two patients with IBD, one patient with CD and one patient with UC. The identity of the *C. concisus* isolates was confirmed by bacterial morphology (small curved and spiral rods), Gram stain (Gram negative) and sequence of 1200 bp 16S rRNA gene (100% similarity to the known *C. concisus* in GenBank).

### Sequences accession numbers

The accession numbers of the sequences of the PCR products submitted to GenBank were JN544934-JN545008.

## Discussion

This study aimed to investigate the prevalence of *C. concisus* in adult patients with CD and the possible preferential colonization sites of *Campylobacter* species in the human intestine; by examining the presence of *Campylobacter* species in 301 intestinal biopsies collected from 28 patients with IBD and 33 controls using PCR-sequencing and *Campylobacter* cultivation.

The high positive rate of *Campylobacter* genus-PCR and intestinal prevalence of *C. concisus* in adult patients with CD and UC observed in this study are consistent with our previous findings in paediatric CD and the findings by Mukhopadhya *et al* in adult UC [13], [14], [15]. A further finding of this study is the increased prevalence of *C. concisus* in the proximal large intestines (descending colon and rectum) of patients with IBD as compared with the controls (Table 4). In adult patients with CD, only biopsies collected from the descending colon showed a significantly higher prevalence of *C. concisus* as compared with the controls (Table 4).

Different *Campylobacter* species may have preferable intestinal colonization sites in their hosts. For example, a study from Inglis *et al* examining the colonization of *C. jejuni* and *Campylobacter lanienae* in asymptomatic beef cattle, *C. jejuni* was found to colonize the proximal small intestine whereas *C. lanienae* was detected primarily in the caecum, descending colon and rectum [19].

In individuals without intestinal inflammation, biopsies collected from ileum, caecum, descending colon had a similar *C. concisus* positive rate and the rectal biopsies had a lower *C. concisus* positive rate (Table 2). However, in patients with IBD, a higher prevalence of *C. concisus* in the proximal large intestine (descending colon and rectum) was detected (Table 4). It is not entirely clear why *C. concisus* was more prevalent in the proximal large intestine of patients with IBD, particularly in descending colon of patients with CD (Table 4). It is possible that this may relate to the fact that *C. concisus* requires hydrogen enriched microaerophilic atmosphere for growth [2]. In the human intestine, hydrogen is produced by bacterial flora through fermentation of unabsorbed carbohydrates; previous studies showing that 99% of hydrogen in the intestine is produced in the colon [20]. The amount of hydrogen produced in the intestine is affected by food type and intestinal bacterial composition [20], [21]. It may be that the microenvironment of proximal large intestine in some individuals is more suitable for *C. concisus* growth. Whether the high prevalence of *C. concisus* in the proximal large intestine of patients with IBD is a primary event or secondary to the disease is not known. The finding in this study that the prevalence of *C. concisus* in newly diagnosed patients is similar to that of the relapsed cases suggests that the high prevalence of *C. concisus* in patients with IBD is likely a primary event.

The finding that *C. concisus* has a preferable intestinal colonization site (the proximal large intestine) in patients with IBD suggests that different bacterial species may be associated with IBD occurring at different parts of the gastrointestinal tract. Other evidence from both human and animal studies supports this view. For example, in human studies adherent and invasive *Escherichia coli* has been found to be associated with ileal CD only [22]. Furthermore, antibiotics used to treat patients with IBD were effective only in a subgroup of patients [23]. In animal studies, IL-10 ^-^/^-^ mice developed caecal inflammation when monoassociated with *E. coli* but distal colitis when colonized with *Enterococcus faecalis* [24].

Whether *C. concisus* detected in patients with IBD has contributed to the pathogenesis of the disease requires further investigation. *C. concisus* is a bacterium with great diversity; which has been demonstrated by various research groups using different methodologies [25], [26], [27], [28], [29]. Intestinal *C. concisus* strains have been shown to be able to induce production of IL-8 in HT-29 cells and some *C. concisus* strains were invasive to Caco2 cells [30], [31]. In addition, the presence of bacterial virulence factors such as phospholipase A2 and a cytolethal distending toxin (CDT)-like toxin in some *C. concisus* strains suggest that some *C. concisus* strains may have the enteric pathogenic potential [32], [33].

Examination of prevalence of *C. concisus* in patients with gum disease by Macuch and Tanner has revealed an interesting relationship between *C. concisus* and oral mucosal inflammation [5]. In their study, Macuch and Tanner found that the isolation of *C. concisus* from subgingival plaque samples of patients with initial periodontitis was greatly higher than the controls. However the isolation rate of *C. concisus* in patients with established periodontitis was greatly reduced in comparison with the healthy controls [5]. These results suggest that *C. concisus* may be only associated with mild oral mucosal inflammation. A more severe inflammatory microenvironment such as the established periodontitis is certainly no longer a favourable environment for *C. concisus*. We have observed a similar phenomenon in patients with CD. In our previous study in a paediatric population, we found that biopsies taken from macroscopic normal area near the inflamed area had higher *C. concisus* detection than biopsies taken from the centre of the severely inflamed area [13]. Patients with UC included in this study had mild to moderate disease severities (Table 1); we therefore were unable to examine the prevalence of *C. concisus* in patients with severe UC.

These data suggest that the role of *C. concisus* in the pathogenesis of IBD, if there is any, would be most likely to facilitate the establishment of the inflammation in the early stage of the disease or to promote inflammation from a mild form to a more severe form. Recently, we found that *C. concisus* has the ability to modulate the gut mucosal immune system through upregulation of the intestinal epithelial expression of Toll-like receptor (TLR)-4 (unpublished data). The low level intestinal epithelial expression of TLR-4 is one of the mechanisms allowing gut mucosal system to maintain its tolerance to commensal intestinal bacteria flora [34]. Accumulated evidence suggests that some intestinal commensal bacterial species are involved in the pathogenesis of IBD [11]. Perhaps the increased intestinal expression of TLR-4 induced by *C. concisus* has upregulated responses of the gut mucosal immune system to some intestinal commensal bacterial species otherwise it would tolerate. This hypothesis requires further investigation.

Despite the high prevalence of *C. concisus* detected in patients with IBD, the amount of *C. concisus* DNA in the intestinal biopsies was generally low. An initial examination of 20 biopsies collected from 5 patients with CD using direct *Campylobacter* genus-PCR revealed low positivity. Given this, we decided to use a nested PCR method to amplify the 16S rRNA gene of universal bacteria and then use *Campylobacter* genus-PCR. The nested PCR has greatly increased the detection rate of *Campylobacter* species in biopsy samples. It is likely that the preparation procedure for colonoscopy, which involves induction of severe diarrhoea, may have contributed to the low number of *C. concisus* in the biopsies.

In addition to detection of *C. concisus* from intestinal biopsies by PCR, we have isolated *C. concisus* from intestinal biopsies of one patient with CD and one patient with UC.

Some other *Campylobacter* species detected in this study have been shown to be clinically important. However, the prevalence of these *Campylobacter* species in patients with IBD was low and not significantly different from that in the controls (Table 3).

In summary, in this study we detected a significantly higher prevalence of *C. concisus* in colonic biopsies of adult patients with CD as compared with the controls and isolated *C. concisus* from intestinal biopsies of adult patients with IBD. Furthermore, we found that *C. concisus* preferentially colonizes the proximal large intestine of patients with IBD. These results suggest that future studies are needed to investigate the possible involvement of *C. concisus* in a subgroup of human IBD. To our knowledge, this is the first report of the association between adult CD and *C. concisus*; the first study of the preferential colonization sites of *C. concisus* in the human intestine; and the first isolation of *C. concisus* from intestinal biopsies of adult patients with IBD.
